# Individual Choice and Risk: The Case of Higher Education

**DOI:** 10.1177/0038038512444814

**Published:** 2013-04

**Authors:** Malcolm Brynin

**Affiliations:** University of Essex, UK

**Keywords:** Beck, British Labour Force Survey, higher education, pay, risk

## Abstract

The expansion of higher education raises the risk environment for school-leavers as more occupations become partially graduate with the result that occupational signals are fuzzy. This makes the educational decision more difficult and more risky, especially with more of the cost of higher education being transferred to the individual. After a discussion of the nature of risk, derived from Beck, and of the role of government policy and of economics in obscuring this, the analysis uses simple quantitative techniques, based on British Labour Force Survey data, to demonstrate the increased fuzziness of graduate work. It is also shown that a rising proportion of graduates receive only average pay, thus raising the risks associated with educational investments even further.

## Introduction

The expansion of higher education has allowed a substantial proportion of young people – in some countries a majority – to obtain a university degree. Yet while young people stand to benefit from this they are also, in Beck’s terms (1992), exposed to greater risk. Most obviously, as the costs of higher education are increasingly shouldered by the student, especially in the UK, the danger of financial loss also increases. The calculation of risk itself becomes more risky; as costs rise more is at stake. However, for Beck, risk is a social parameter, not only an individual calculation. In the case of higher education, expansion has blurred the boundary between graduate and non-graduate work and thus altered the risk environment. Knowing what is a graduate job is surely an important factor in the decision whether to participate in higher education.

It is unlikely that many young people calculate the economic value of education relative to an expected career. They are likely instead to have a notion of a ‘good’ job, which would partially be based on some (often vague) idea of expected pay, but also on the job’s prestige and the skills it requires. The latter are particularly important but are difficult to assess when graduate employment is rapidly increasing. While some occupations have become wholly graduate and others remain wholly non-graduate, many occupations are now partially graduate, producing unclear occupational choices. This is not the sole cause of a lack of defined occupational norms, as occupational boundaries are altered by many factors, including rapid technological change (Purcell, 2000), but whether a job is perceived to be graduate is likely to be critical to the individual’s decision to go to university, and this occupational norm is becoming less certain.

This article investigates the parameters of this growing risk through descriptive quantitative analysis of the proportion of employees in occupations comprising graduates and the pay they can expect to receive. It is shown that an increasing percentage of graduates enter jobs which are neither clearly graduate nor clearly non-graduate, so that many graduates compete against non-graduates economically while often they will be doing the same type of work. The main test is the proportion of graduates receiving average pay. Has this risen or fallen? Before this analysis the article argues that the risk environment is partly created but also masked by uncritical policy arguments and apparent social-scientific evidence in favour of individual investments in higher education.

## Education and the Risk Society

Belief in the value of education makes it difficult to recognise that education is part of the risk society. There has certainly been a long-term critique of education within sociology, and to a far lesser extent economics (e.g. Bowles and Gintis, 1976), but this has been directed mostly at its maldistribution and its link with social inequality (e.g. Bourdieu and Passeron, 1990; Halsey et al., 1980; Jencks, 1972; Shavit and Mueller, 1998), which with the expansion of education and growing strain on public budgets has since then mutated into a firm policy concern (e.g. within Britain: DBIS, 2011a, 2011b; DES, 2003: 17). Rarely, though, has the value of education itself been questioned (with extreme exceptions such as Illich, 1970). While access to higher education is still unequal, and while unequal access might extend to inequality in forms of delivery (Ainley, 1994; Trowler, 1998), the expansion of higher education is nevertheless spreading risk to an increasingly large proportion of the young population. For Beck risk is diffuse (2000a: 3).

Beck’s analysis is suggestive but not conclusive, however. Its often pessimistic language can sometimes be easily contradicted. For instance, in arguing for an individualisation of risk in employment (1992: 139–50, 2000a), Beck relies on evidence of a trend increase in flexible labour through part-time, temporary contracts, and reduced job tenure, but this evidence is not strong enough to confirm a major shift. It is at best partially true, varies greatly by country, and is subject to contradictory trends (Gallie, 1996; Green, 2006; Muffels, 2008). Yet there is other evidence in favour of the idea of the individualisation of risk in employment, which Beck does not cite and which has education rather than employment as the central problem. One indicator is the large proportion of employees overqualified for the work they do, which varies over time and across countries from about 20 per cent to even 50 per cent (Borghans and De Grip, 2000; Büchel et al., 2003; Hartog, 2000). This evidence shows that overqualification lowers returns to education. It is also associated with reduced job satisfaction and high occupational turnover as people find themselves in the wrong job or even wrong type of job (Longhi and Brynin, 2009; Parrado et al., 2007).

In the case of graduates, even in the early 1990s concern was expressed at narrowing wage differentials between graduates and non-graduates (Murphy, 1994); overqualification amongst graduates – that is, graduates doing non-graduate work – is common (Dolton and Vignoles, 2000; Purcell et al., 2005). As education is not fully utilised, on average this gives rise to lower wages than would otherwise be the case (Brynin, 2002). Pearson, using data on graduate recruitment, talks about the ‘demise’ of the graduate labour market as a decreasing proportion of graduates have managerial or professional jobs. ‘For the majority [of new graduates], moving into employment is a slow transition with many experiencing several years of turbulence and having to compete for jobs with non-graduates …’ (2006: 76). Chevalier and Lindley find that overqualification increased during the expansion of higher education in the UK in the 1990s by around one-third and argue that as a result of oversupply ‘non-traditional graduate jobs have been upgraded to make use of the additional supply of graduates’ (2009: 333), though only a proportion of the overqualified are nevertheless dissatisfied with the work they do. Primarily but not wholly in the UK there has also been a long-term pursuit of a general rather than vocational education (e.g. Sanderson, 1999), which exacerbates this problem. Mason notes an increasing reliance on graduate employment in Britain relative to higher intermediate vocation qualifications and that this is partly driven by excess supply of individuals with degrees; this causes employers to substitute graduates for non-graduates even where employers ‘are unable to identify any improvement in performance …’ (2000: 18).

Overqualification, low pay and occupational turnover are all indicators of an increasingly poor fit between the supply of and demand for graduates. This misfit is itself an indicator of the growing risk environment that school-leavers face.

## The Manipulation of the Risk Environment

Beck points to the role of science in creating risk, then masking it, a possibility which arises from the fact that an individual cannot generally measure this. In the case of pollution, for example, we rely on public, not private information. Individuals no doubt know that the educational strategies they adopt have uncertain outcomes, but risk is further built into the system precisely through the attempt to minimise it. One example is ‘assistance’ provided by university league tables. Through operations such as aggregation of indicators or treatment of missing data, as well as the weightings given to individual components, these are liable to produce contrived and misleading outcomes (Davies, 1997; Goldstein and Spiegelhalter, 1996; Marginson, 2007; Oswald, 2007). There is some evidence that would-be students in fact pay little attention to such rankings (Eccles, 2002), and also, where they do, that the advantage is skewed by social class or similar factors (Clarke, 2006), but the point here is that such information appears to reduce the risk environment while not reducing the actual risk.

The ‘numbers game’ helps structure the political and ideological debates around higher education (Davies, 1997: 50). Policy-makers, for instance, might see the expansion of higher education as a solution to the UK’s long-standing skills deficit, but from the individual (and perhaps national) point of view support for expansion of high-quality vocational training might be both more useful and less risky. One possible interpretation is that individuals are being asked to risk financial loss over their careers in response to ongoing policy failures. A policy document might clearly state that ‘The benefits of higher education for individuals are far-reaching’ then vaguely qualify this: ‘On average, graduates get better jobs and earn more than those without higher education’ (DES, 2003: 4), yet the qualifier ‘on average’ is critical. This powerful message masks the risks individuals face when they perhaps decide they have no choice but to invest; it also masks the role of the state in spreading the risk environment. Other policy documents are less inhibited than the above.

Along with what to study, one of your biggest questions about higher education will be how to pay for it. There’s more financial help available than you might think.

What’s more, 94% of students agree that university is a good investment. … The qualifications you earn can help you get a better job with much better money – in fact, over the course of your working life, if you’ve got an undergraduate degree, you can expect to earn, on average, comfortably over £100,000 more than someone similar with two or more A levels, net of taxes and in today’s valuation. (DBIS, 2009)

With the ‘risk regime’, ‘people are expected to make their own life-plans, to be mobile and to provide for themselves’ (Beck, 2000a: 70). The prediction that a graduate job is ‘necessary’ becomes a self-fulfilling prophecy because the demand for higher education changes the structure of occupations, making them increasingly graduate and therefore increasingly desirable. ‘Everyday life thus becomes an involuntary lottery of misfortune … It has become almost impossible *not* to take part in this raffle …’ (Beck, 2000b: 217). Are students ‘involuntary captives’ (Riesman, 1980: 90)? The issue here is not whether there is more risk than in the past but that risk is increasingly unavoidable.

Economics, in contrast to much sociology, is almost cheerfully optimistic, tending to see in the above problems simply ‘random noise’ rather than a central problem, as for instance in criticism of the idea that overqualification is an indicator of market failure as opposed to a temporary career adjustment (Sloane, 2003). The market is not perfect but is generally held to work at least approximately. This faith is perhaps most clearly visible in the concept of human capital. While this has its critics (e.g. Manski, 1993) it is a resilient idea. Graduates earn more than non-graduates and will over a lifetime more than recoup the costs of their investments. Prospective graduates perceive this to be the case and so invest in their own ‘human capital’, much as an industrialist invests in machinery. Most empirical studies confirm the theory’s results (e.g. Harmon et al., 2001). This is the case even when they start out from different theoretical premises such as signalling theory (Spence, 1973). There are imperfections: for example high-ability children of low-income parents are unable to finance their education, but then the problem is simply one of imperfect financial markets, therefore not fundamental (Checchi, 2006: 87).

Human capital theory can, however, be taken to be an example of Beck’s ‘manufactured uncertainties’ (2000b). That young people calculate costs against benefits even roughly has always been a figment of economists’ imaginations, acknowledged even within the discipline. ‘Having witnessed the struggles of econometricians to learn the returns to schooling, I find it difficult to accept the proposition that adolescents are endowed with this knowledge’ (Manski, 1993: 49). The prospective student has, according to economists, to estimate the ‘purchase’ cost of education itself plus maintenance costs, foregone earnings while out of work studying, interest costs on any loans, and against this the likely earnings, discounted to take inflation into account. From the individual’s point of view the process is often akin to fortune-telling, and yet the pressure to work something out increases; rising costs force the calculation of the incalculable.

Young people might have only vague career aspirations. One study in Britain found that during post-16 education only 21 per cent of students had ‘definitely decided’ on a future career (Dearing, 1996: 49). Even the decision whether or not to remain in education post 16 is often subject to change; while a large core of students maintain fairly stable plans this masks not insignificant year-on-year fluctuation (Brynin and Bynner, 2003; Croll, 2009). At a very early age career aspirations tend to be unrealistic. While these become more focussed over time, even by the age of 17 a large proportion of students do not view future work in terms of future earnings, ‘with few making rational decisions based upon their skills’ (Morris et al., 1999: 67). While governments assume that young people are human capitalists, using a large qualitative sample Ball et al. find not only that a substantial proportion eschew planning but that ‘the decisions and strategies of those who plan do not appear to be solely or even primarily related to the calculation of economic returns’ (2000: 18). Education is simply one element of the process of forming, or opposing, identities. The educational decision is especially difficult in homes where there is no previous experience of higher education; it is often achieved after a process of uncertainty, shifting ideas, emotional stress, and inputs from a range of family viewpoints. Young people in these situations tend to follow step-by-step paths through education rather than jumping straight from school to a degree course at university (Christie, 2009; Forsyth and Furlong, 2003; Green and Web, 1997; Thomas and Quinn, 2007: 48–66). Payne’s review of post-compulsory choices in particular points to difficulties for children of working-class parents where the decision to go to university is seen as fraught with cultural, emotional and economic risk (2003). Class background no doubt plays a role here, dividing risk into several types. Those with a higher class background are likely to see risk in terms of failure to maintain class position (Breen and Goldthorpe, 1997), which is therefore additional to the risk of financial loss, though at the same time the latter will be limited in wealthier households; for those with a less well-off background both financial resources and expectations are likely to be lower, with the result that for some it might be more rational not to risk at all. In between these two extremes risk is considerable.

People do not behave in the way that human capital theorists suppose. Just as critically, the empirical evidence these theorists use to justify the theory is misleading. This evidence consists of the stability of the ‘graduate premium’ – the pay advantage to having a degree. This has generally held up fairly well despite educational expansion, suggesting a rising demand for skills, which produces appropriate pay signals (e.g. Blundell et al., 2000; Harmon et al., 2001; McIntosh, 2005). There does in fact seem to be some *decline* in the value of a degree, if calculated differently (Brynin, 2002; Elias and Purcell, 2003), but more important is that the traditional analysis of the returns to education generally takes no account of distributions. What little work exists on this in fact suggests that returns are higher at the top end of the wage distribution (Harmon et al., 2001: 14). In other words, some graduates earn huge premia, but this means that the calculation of ‘average’ premia contains considerable redundancy. Many professional people, for instance in medical occupations, or those working in finance, earn very substantial salaries. Their pay raises the apparent average return to a degree very considerably, while perhaps the bulk of graduates, a proportion of whom will be doing work that can be undertaken by non-graduates, earn little more than non-graduates, and some might earn less. For these people the returns to a degree, especially as the cost of education rises, is uncertain and possibly negative. It is distributions that we should deal with.

## Methods

The analysis has three objectives. The first relates to the idea of an occupational norm. What is a typical graduate job and what proportion of employees can be considered to be clearly in neither graduate nor non-graduate work? The risk environment derives from the tendency of the system to produce confusing signals. Occupations are associated with a variety of signals, which surely include the skills expected for the job. These are defined here by the percentage of graduate employees in an occupation. This operates as a visible norm, a signal to potential entrants saying whether the type of job they want is graduate or not. While the percentage of graduates in occupations is rising, so that the norm is generally positive, this norm is also uncertain. This is demonstrated simply through examining what proportion of occupations can be considered to be in this ‘grey’ zone.

Second, when we look at the distribution of pay, for what proportion of employees is a degree helpful? For an economist the measure of risk is really the graduate premium – the gap between the wages of graduates and non-graduates; however, this ignores distributions. A proportion of graduates do well, others far less well, and it is the proportion placed at risk which is important. The analysis therefore compares the wage distributions of graduates and non-graduates.

Third, is getting into a higher class the only objective of being a graduate or does this still mean that a graduate can be doing a poor job? Class alone cannot tell us enough about risk. Does achieving a ‘good job’, that is, not only a ‘high-class’ job but within this one that also pays well, depend on being a graduate? This analysis introduces occupational class, using the British NS-SEC^1^ (Rose et al., 2005).

The analysis is based on data from the British Labour Force Survey (LFS), covering 1993 to 2008 (starting in 1993 as this is the earliest date for which wage information is available). Wage data are deflated so that a pound is worth the same whatever the year and is the hourly wage in all cases. The analysis covers men and women aged 16 to 60 who work at least 10 hours a week. Those with extremely high and low wages (under £1 or above £80 per hour) are excluded. For additional analysis of generational class effects the British Household Panel Study (BHPS) is used.

## Results

### The Risk Environment

It was argued above that a degree is increasingly necessary for those wishing to do well in their careers economically. Work by economists supports this belief, as do government reports and much media coverage. The risk environment impels young people into higher education, and so the government’s claim becomes a self-fulfilling prophecy: more people enter work as graduates, so more jobs seem to be graduate jobs. At the same time, however, the graduate norm is uncertain. Arbitrarily defining a range of graduate density (the percentage of employees in an occupation who are graduates) of between 10 and 40 per cent as ‘indeterminate’, that is, neither graduate nor convincingly graduate, then the proportion of all employees in this zone of uncertainty increased over the period from less than 22 per cent in 1993 to 38 per cent in 2008. While some occupations are becoming entirely or at least largely graduate, others are moving from non-graduate to marginally graduate or from marginally to partly graduate. However, few graduates work in manual occupations. If we look only at non-manual occupations, these last two figures rise to around 37 per cent and 60 per cent respectively. By 2008 getting on for two-thirds of non-manual employees worked in occupations which cannot be defined as clearly graduate or non-graduate. It is not possible to produce percentages for specific occupations across the entire period as a result of the change to the coding system in 2000 (from SOC90 to SOC2000), but in some cases this is possible. For instance, ‘scientific technicians’ were 17.2 per cent graduate in 1993, 20.6 in 2000 and 25.2 per cent in 2008; ‘health associate professionals’ such as nurses, midwives or dispensing opticians were 13.5 per cent graduate in 1993, 22.4 per cent in 2000 and 30.8 per cent in 2008; and, at a lower level of graduate employment, ‘numerical clerks and cashiers’ (accounts clerks, counter clerks and debt collectors) were 6.8 per cent graduate in 1993, 9.7 per cent in 2000, and although relabelled slightly thereafter as ‘administrative occupations: finance’ (credit controllers, accounts clerks and counter clerks), 13.7 per cent in 2008.

Many occupations are neither clearly graduate nor non-graduate, thus offering risky signals. The risk itself can be quantified in terms of pay. More and more school-leavers have to become graduates in order to earn only average pay. To define the latter the arithmetic mean is used, thus discounting the fact that the wage distribution is highly skewed (many people receive low pay, a small number are highly paid), but this average is treated broadly as any (hourly) wage between 30 per cent below and 30 per cent above the precise mean. Looking only at non-manual work, in 1993 over 24 per cent of graduates were in this wage band. By 2008 the figure was over 34 per cent. To earn a merely average pay it is increasingly necessary to be a graduate; currently, average pay requires one-third of employees to be graduate. It is quite possible that in another 15 years about half of all those on average pay will be required to be graduates. The bar is not rising, it is sinking.

The increase in graduations can be interpreted as a success story, but it also leads to a growing grey area where it is no longer clear what is and what is not a graduate job, or whether a graduate job produces more than average pay. The risk environment for prospective students is increasing.

### The Distribution of Risk

**Figure 1.**
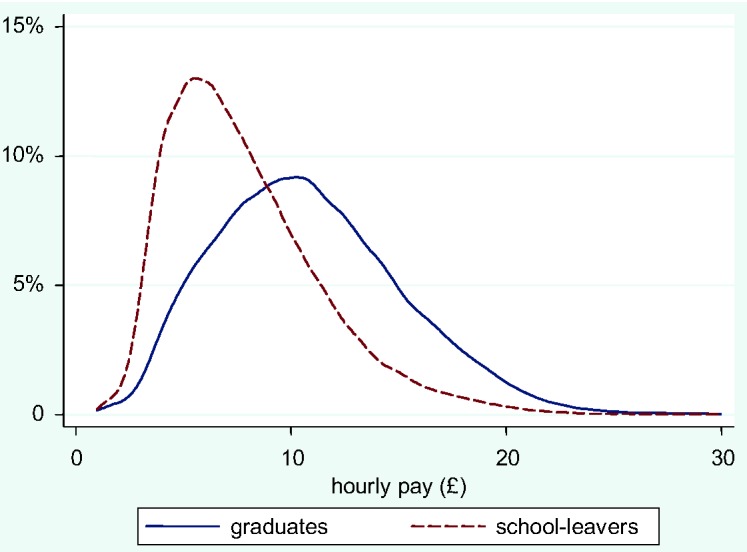
Distribution of hourly pay for graduates and school-leavers with A-levels.

A central concept in the above discussion is the importance of distributions. There is little point in showing that on average graduates earn more than non-graduates if the pay of the two groups nevertheless overlaps considerably. The arithmetic means are £10.90 per hour for graduates and £7.90 for their nearest competitors, those with an A-level or equivalent. On average being a graduate pays. However, the distributions greatly overlap. This is demonstrated in Figure 1, which, averaging across all years of the LFS, shows the percentage distribution of pay for graduates (the solid line) against school-leavers with only A-levels (broken line). The (modal) average pay for the former is clearly higher, as seen in the positions of the peaks of the two curves, but a high proportion of graduates earns much the same as A-level school-leavers, so that many graduates benefit little from their degrees. Getting a degree is a gamble.

The pay distribution can be seen in more detail in the first two columns of Table 1, which, building on the previous analysis, breaks this into the following categories:

**Table 1.** The distribution of graduates across wage bands.

	All employees		Non-manual employees	
*Wages*	*1993*	*2008*	*1993*	*2008*
Below average	6.6	10.3	8.2	27.1
Average	28.7	38.0	43.9	49.8
Above average	64.7	51.7	47.9	23.1
*Total %*	*100*	*100*	*100*	*100*

1. Below average: Less than 30 per cent below mean pay of all employees (graduate or non-graduate)
2. Broad average: between 30 per cent below and 30 per cent above the precise mean
3. Above average: More than 30 per cent above the mean.

To maximise sample size, years are now grouped into two periods: the last years of the old century and the first of the new.

The first column unsurprisingly shows that graduates predominate in the above-average wage group while only a small percentage are below average. So far so good. However, the situation is dramatically worse in the later period with the percentage of graduates in the higher paid group plummeting while the percentage in the low-paid group rises considerably.

Clearly, graduates would expect to be doing non-manual work and to compare their pay to that of other non-manual employees. Based on average pay for *non-manual* employees the final two columns show a more equal distribution of graduates across the wage groups, but in the later period there is an even bigger drop in the proportion in the higher pay category and a big increase in the lowest, while around half are in the average pay band.

To look at this the other way around, instead of showing the percentage of graduates who fall into each wage band, Table 2 examines the percentage of each band comprising graduates. Over time this is likely to increase at all three levels because the percentage of graduates in employment has increased. The final row of Table 2 shows that the percentage in employment overall increased from 14.9 per cent to 22 per cent. The biggest increases were in the highly paid and average paid groups. Taking non-manual workers only we see a much more serious position, with a small increase in the highest paid group but an alarming increase in the low-paid group; the percentage of graduates in this nearly doubles in size. So we can see that the increase in graduations has led to a substantial increase in poorly paid graduate employment in non-manual work. The graduate explosion is associated with an increased entry into non-manual work, but more specifically into low-paid non-manual work.

**Table 2.** The percentage of graduates within wage bands.

	All employees		Non-manual employees	
*Wages*	*1993*	*2008*	*1993*	*2008*
Below average	2.8	7.1	6.3	15.6
Average	10.3	18.8	24.2	34.3
Above average	40.9	48.2	51.7	57.2
*Average %*	*14.9*	*22.0*	*24.8*	*34.5*

## Risk, Higher Education, and the Service Class

There has been much argument over the relationship of class of origin to both educational achievement and subsequent class outcomes. Although evolving into more complex arguments over the role of cultural capital in these relationships (e.g. Harrison and Waller, 2010), class distributions surely remain important. Most evidence seems to suggest that class background is important to obtaining entry into higher education but subsequently confers some though often little further advantage, (Goldthorpe, 2007). These two stages seem important in other ways. This analysis has been undertaken here using the BHPS. As this is not central to the argument the results are not shown, but they confirm that the higher the father’s class, the more likely a child will graduate and then enter the service class, but father’s class has little association beyond this with either doing a typically graduate job or high pay. In fact, it also appears that father’s class is decreasingly associated even with educational success.

For economists the graduate premium is an indicator of the success of human capital theory. The premium has held up over time, suggesting not only that the theory is correct but that demand for skills is continuously rising, in support therefore also of the ‘technology bias thesis’. Sociology has a different way of looking at this but with somewhat similar expectations. Essential at least to some views of occupational class, the rising demand for skills leads employers to offer contracts to employees which are intended to retain the loyalty of their more skilled employees. This generates a class of employees whose contracts encourage continued service even while their skills give them considerable autonomy, as exemplified in part by increasing graduations within this group. The class result seems a parallel to the arguments of economists: a degree is increasingly necessary to entry into a ‘good job’. In 1993 graduates comprised 52 per cent of employees in the upper service class (USC), 24 per cent of the lower service class (LSC). By 2008 these figures were 56 per cent and 35 per cent, demonstrating that in the USC an upper ceiling is perhaps being reached while there is a vigorous growth of ‘professionalisation’ (if we say that graduate work is an aspect of being a professional at least) in the LSC.

Despite the rises in the size of the service class and their basis in graduations, it is by no means the case that entry into the service class is necessarily worthwhile economically. Pay varies enormously within classes (Savage, 1992: 77–8; Van de Werfhorst, 2007) but even in terms of averages the advantage is less clear than it was. Table 3 shows the average pay in the three ‘top’ classes where graduate employment is common. Because distributions are so important, both the median and the mean are shown. Looking at the median, which gives the average at the midpoint of the distribution, there is a clear wage hierarchy across the classes, both amongst non-graduates and graduates while graduates also earn more than non-graduates in all classes. Further, this average increases over time (with the exception of graduates in the intermediate class), though it is of note that the increase is stronger amongst non-graduates. However, while the class hierarchy and difference between graduates and non-graduates applies also when the mean is calculated, the increase over time for graduates is at best negligible and even slightly negative.

**Table 3.** Average pay within the higher and lower service class and intermediate class (£ per hour).

	Non-graduate employees				Graduate employees			
	Median		Mean		Median		Mean	
	*1993*	*2008*	*1993*	*2008*	*1993*	*2008*	*1993*	*2008*
USC	9.5	11.0	10.2	11.2	11.9	12.5	12.7	12.6
LSC	7.1	8.3	7.7	8.8	10.1	10.7	10.6	10.9
Intermediate	5.3	6.2	5.8	6.8	7.3	7.2	8.1	7.8

**Figure 2.**
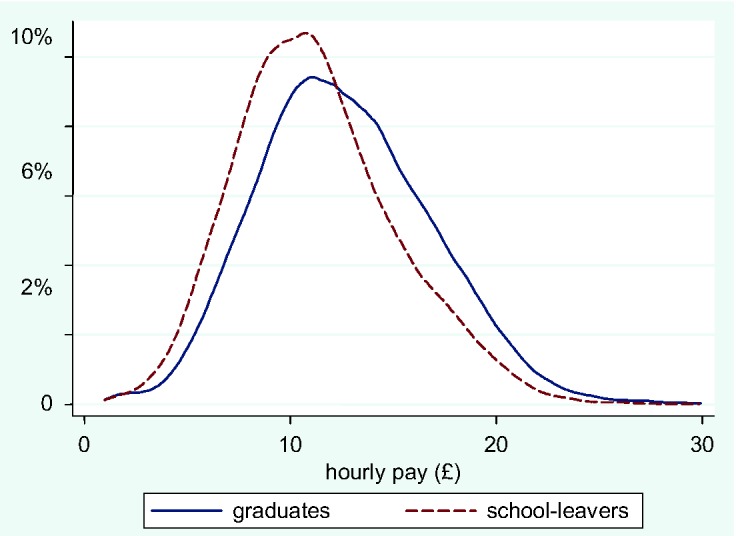
Distribution of hourly pay for graduates and school-leavers with A-levels (upper service class).

This therefore does not mean that all graduates have done badly; rather, the ranks of the graduate sector have been so swelled that they now include many poorly paid people – they affect the mean far more than the median. Any class, and especially the simple label ‘service class’, masks very substantial heterogeneity in terms of wages. To return to the analysis shown in Figure 1, which compared the wage distribution of graduates in employment to that of school-leavers with A-levels, the same is undertaken in Figure 2 for employees in the upper service class only. Here the distributions overlap very considerably. It makes little difference to the majority of people in the USC whether they have a degree or not; a degree helps get a job in the USC but confers little further wage advantage.

Finally, bringing the two forms of analysis together, results from two regressions are shown in Table 4 for the effects of education. The first four columns give results for the effects of education on (log) wages (using ordinary least squares, not showing controls). The figures can be interpreted as roughly the percentage effect on actual wages of having either a degree or A-levels (or equivalent), compared to having a low (or no) education. Thus, in this comparison, a degree in 1993 increases hourly wages by about 52 per cent, much higher than the effect for A-levels, so again on average a degree pays, now controlling for other factors. However, by 2003 the degree effect diminishes slightly and by 2008 substantially while that of A-levels rises, reducing the gap from nearly 40 to less than 30 percentage points, thus again indicating the increasing risk of educational investments. The next four columns look at entry into the USC (including job-level controls such as industrial sector because promotion into a managerial job might in some cases depend on this). Here the figures are the effect on the odds of entry. In 1993 a degree massively raises the odds of having a job in the USC relative to those with little education. If a degree is not an entry requirement it helps a great deal. However, the benefit declines over time and by 2008 had decreased considerably.

**Table 4.** Effects of education on log hourly wages (OLS) and employment in upper service class (logistic regression).

	Hourly wages				USC employment			
	*1993*	*1998*	*2003*	*2008*	*1993*	*1998*	*2003*	*2008*
Degree	0.52	0.52	0.50	0.46	20.20	16.96	15.79	11.73
A-level	0.14	0.23	0.20	0.19	2.84	2.65	2.57	2.34
*(Pseudo) R* ^*2*^	*.44*	*.45*	*.44*	*.40*	*.31*	*.31*	*.30*	*.26*
*N*	*18325*	*55806*	*45603*	*38543*	*18135*	*55316*	*44534*	*37620*

*Notes*: Controls not shown: age, age squared, gender, ethnicity, job tenure, industry, region, proportion feminine in occupation. All results significant to *p*<.001.

## Concluding Discussion

Sociology in its early days tended to be a pessimistic subject, seeking to explain a perceived loss of community, the depredations of industrialisation, and new, profound forms of inequality and oppression. While the subject subsequently became more expansive and optimistic, for instance through the writings of Parsons, indicating that social processes tended to maintain the social structure, or Bell, for whom there are new bases of social progress, a second wave of despondency has developed, especially through the concept of the ‘risk society’ popularised by Beck. In contrast to Bell’s knowledge society, and also to the underlying optimism in much thinking by economists, we have an explosion of risk, uncertainty, and ‘unknowing’. Perhaps most disturbing is that we can transfer this pessimism to an understanding of higher education, the development of which is perhaps one of modern society’s greatest achievements.

In the words of an unemployed graduate reported in a newspaper report on youth unemployment in the UK (*The Independent*, 11 October 2011):

… our generation was given a message that there would be a world of opportunities waiting for us after university – but what are these opportunities? Mostly just internships that don’t pay for the basics of living. It’s a tough time to be young.

Government cuts and recession have recently added to this, but also important is that while a degree is more and more a prerequisite for a ‘good’ job, paradoxically it is increasingly uneconomic for a substantial proportion of graduates. Many are graduates earning non-graduate pay who can perhaps be seen as paying an economic price for the expansion of higher education encouraged by government. They are what can be called a ‘frictional loss’ required by the system to operate at a high tempo. Individuals cannot and do not calculate the expected returns to their educational investments, not even indirectly, but are led to believe by the assertions of economists and politicians that education pays. The cost of their educational investments can therefore be put up very considerably, raising the risk of failed investments for more and more people. As Halsey (1997) argues, aggregate access to higher education is not so much an economic as a political decision.

This does not mean that young people’s educational decisions are unaffected by costs. This can be the increase in direct costs, the rise in opportunity costs if work alternatives to study become increasingly attractive, or the effects of recession, which lowers income prospects. The latter is possibly the cause of a slowing down in the increase in demand for university places in England from 2008 to 2010 (Campo et al., 2011: 60), and there are now signs that the British ‘natural experiment’ – the trebling of tuition fees – is lowering university enrolment (by about 7% over the previous year), as widely reported in the national media in January 2012 on the basis of figures from the Universities and Colleges Admissions Service. However, far from indicating that individual and instrumental rationality works (because the decision might be based on an instinctive feeling that the higher costs are prohibitive, or even unfair), this suggests an increasing and increasingly risky burden on the decision-making of young people.

Risk is also increasingly affecting institutions. While it is possible to argue that through expansion higher education is changing from being a ‘*premodern* to a *modern* institution’ (Barnett, 1994: 3–4), according to Barnett the increasing reliance on concepts of rationality and performance which this entails also comes at a cost: ‘higher education is being locked into a Weberian iron cage of over-prescriptive rationality, of given ends and of operationalism’ (1994: 5). Institutions of higher education, and within these their professional staffs, are being made more accountable, but one purpose behind this improved transparency is the quest for greater competition between institutions. These often now operate on pseudo-market lines especially in ‘liberal’ countries such as the UK and the USA, visible not only in teaching but in making universities profitable research markets (Washburn, 2005). More ‘corporatist’ countries such as Germany (Kehm, 1999) are now following suit, if more cautiously. We therefore have two apparently contrary processes: on the one hand, increasing rationality in the form of consistent measurement of inputs into and outputs from universities, on the other the growing differentiation and complexity of institutions, degree formats, and courses – all part of the ‘commodification’ of higher education.

That risk now extends to institutions is not surprising. While it has been argued that there is no crisis in higher education, which by and large comprises relatively successful institutions in terms of both organisation and morale (Watson, 2009), massive cuts in government spending have changed this. Both young people and universities are now caught between two government policies, one designed to encourage expansion, itself largely a response to previous failures in educational policy, and the other to encourage contraction as a result of the perceived need to reduce government spending.

## Notes

1. National Statistics Socio-economic Classification.
